# Strategic Uncertainties Surrounding Mosquito-Borne Disease Policy-Making in the Netherlands: A Game Theoretic Analysis

**DOI:** 10.34172/ijhpm.9091

**Published:** 2026-06-29

**Authors:** Henk Broekhuizen, Femke Bekius, Pauline A. de Best, Marleen Bekker

**Affiliations:** ^1^Health and Society Group, Department of Social Sciences, Wageningen University and Research, Wageningen, The Netherlands.; ^2^Department of Epidemiology, Health Promotion, and Policy Advice, GGD Twente, Enschede, The Netherlands.; ^3^Department of Methodology, Nijmegen School of Management, Radboud University, Nijmegen, The Netherlands.; ^4^Department of Viroscience, Erasmus Medical Center, Rotterdam, The Netherlands.; ^5^National Institute for Public Health and the Environment (RIVM), Bilthoven, The Netherlands.

**Keywords:** Evidence-Based Policy, Governance, Public Health Policy, The Netherlands, Zoonosis, Mosquito-Borne Disease

## Abstract

**Background:**

One Health decisions are challenging due to substantive and strategic uncertainty. This study aims to map and interpret strategic uncertainties surrounding a specific One Health issue (mosquito-borne disease [MBD] policy) in the Netherlands.

**Methods:**

We used a game-theoretic framework to identify games from 26 interviews with key actors about strategic uncertainty. For each game we identified the context (where the game is played), the actors (who is playing), the content (what are the issues and outcomes), and the process (how the game has developed and what strategies were employed). Games with the same set of actors and overlapping content were clustered together.

**Results:**

We identified 15 games, centered mostly around trade-offs between domains (human health, animal health, and the environment) and dilemma’s inherent to prevention policy. We identified three game clusters. *Multi-level governance* forms the context around MBD policy. It consists of a game involving issues around climate adaptation, infectious disease policy, and biodiversity. This game is played across time and hierarchical levels (local, regional, national), giving rise to cascade dynamics. In this governance context, actors deal with *responsibilities around invasive mosquito control*. This second cluster is characterized by a dilemma between acting (and bearing the cost) and waiting for someone else to act; and a game represented by a central notification hub. Finally, the *zoonotic structure* was developed to bridge domains and gives rise to a various games representing the tension between actors’ own and shared interests, power and information asymmetries, and acting versus not acting for their own benefit.

**Conclusion:**

We argue for an actor-centered approach and more awareness of strategic uncertainties among involved actors in MBD policy to reduce strategic risks inherent in the identified game archetypes. We provide policy recommendations for anticipating, reducing or dealing with the associated strategic risks towards better MBD prevention.

## Introduction

**Key Messages**

**Implications for policy makers**

- Due to climate change and globalization, the risk of mosquito-borne diseases (MBDs) in northern European countries such as the Netherlands is increasing. Involved actors need to negotiate how to deal with this changing and uncertain situation. We investigated strategic uncertainty in this context using a game theoretical lens.
- Identified games are characterized by trade-offs between domains (human health, animal health, and the environment), and the Volunteers Dilemma is inherently present in prevention policy. Strategic uncertainty about other actors’ incentives and priorities in these games can lead to unilateral decisions based on individual interpretations of siloed evidence, potentially resulting in suboptimal outcomes.
- For the formulation of future-proof MBD prevention and control policy it is important to consider not only substantive uncertainties. There is a need for more awareness of strategic uncertainties and an actor-centered approach. Insight into strategic uncertainties could enable actors to collaboratively design new governance strategies that can help to articulate, anticipate, and mitigate strategic risks. The game theoretical concepts provide a way to structure the strategic uncertainty, identify strategic risks, and provide a perspective of action.

**Implications for the public**

Some diseases can be transmitted by mosquitoes. The risk of getting these in northern Europe is low. However, globalization and climate change are expected to increase this risk. Developing ways to mitigate and manage mosquito-borne diseases (MBDs) is difficult because many organizations have to work together. This presents them with strategic uncertainty: they do not know what to do. This paper tries to understand this uncertainty by looking at it with game theory. We provide insight into how these organizations work together and propose ways they can improve collaboration despite differences in how they view the world and what priorities they have.

Mosquito-borne diseases (MBDs) are zoonotic infectious diseases that are transmitted between animals and/or humans by mosquitoes. There is an increasing risk of these diseases in Europe due to global transport, travel and shifting climate zones.^1-3^ In the past few decades several invasive mosquito species have been introduced and established in Europe, mainly in southern Europe but slowly moving north. These, together with endemic mosquito species, have caused local or regional outbreaks of MBDs like Dengue, Chikungunya and West-Nile fever.^4-6^ In the Netherlands both international trade and tourism have influenced the introduction and expected establishment of invasive mosquito species.^7,8^ The increased risk of MBDs due to invasive mosquito species, and the first human West Nile virus cases in 2020, make further context changes likely and call for a rethinking of a previously mainly surveillance-based MBD policy.^9,10^ There is, however, uncertainty about what to do. On the one hand, there is *substantive uncertainty* about how invasive mosquito establishment (and associated disease risks) will evolve and what timing, location, or magnitude of disease outbreaks can be expected.^9,11^ Such substantive uncertainties are gaps in the scientific evidence base around mosquitoes the pathogen they can transmit. On the other hand, there is also s*trategic uncertainty *becausethere are many actors in the Netherlands who are (in)directly involved with MBD policy: a multi-level and multisectoral government and executive agencies, research institutes, corporate actors, the general public, and the media. Each of these actors have different priorities and perspectives based on their respective contexts and (lack of) specialized knowledge. A recent network analysis of interactions between these actors reveals that they collaborate and share information around MBD policy to a limited extent only.^12^ This complicates joint assessment, decision-making and action. Actors then face*strategic uncertainty* about what decisions to take. If strategic uncertainty leads to undesirable outcomes (eg, delays or unilateral action) for all involved actors these will be termed *strategic risks* in this paper.

 Thus, besides substantive uncertainties, actors must also deal with strategic uncertainty. Although the substantive uncertainties around MBDs have been researched or are the topic of current epidemiological and biological research (see eg,^11,13^), strategic uncertainties in the MBD domain remain understudied.^14-17^ The goal of this study is, therefore, to map and interpret strategic uncertainties surrounding MBD policy in the Netherlands. Through this we aim to provide policy recommendations for anticipating, reducing or dealing with the associated strategic risks towards better MBD prevention.

 We adopt a *game theoretic perspective*^18,19^ to investigate and explain the strategic uncertainties of complex decision-making processes. Game theory provides a multi-actor perspective to decision-making revealing actor-specific drivers as well as specific bilateral or multilateral interaction modes between actors. On the one hand, the interactions between actors, actions and decisions explain where strategic uncertainty comes from and how it manifests. Thereby, it allows us to better understand and explain MBD policy dynamics. On the other hand, based on the game theory characterization, strategic uncertainty can be (better) navigated. Meaning, the actions of other actors can be anticipated, and it can help with the design of enabling decision-making processes.^18^ Although we have to keep in mind that rationality in real world decision-making is bounded, and one of the assumptions of game theory is rationality of actors, for these reasons we find it a useful lens for this study. The particular approach used in this study includes game theoretical concepts, namely archetypal *games*. These games are well-studied parts of the public administration and game theoretic literature and support real-world decision-makers in various domains.^20-23^ These games originate from both game theory and public administration science and have been used extensively in various domains other than public health.^18,24^

## Methods

###  Theoretical Framework

 Game theory revolves around *games*, which are situations in which a set of actors make strategic decisions depending on their own objectives and the decisions of other players.^21,25^ These decisions lead to outcomes for each actor individually and for the group of actors involved. In line with previous studies,^21,22^ we have selected seven games based on relevant characteristics of complex decision-making. We refer to Bekius and Meijer^26^ for a more detailed explanation of the selection of the games. Table presents and shortly explains these games. The games’ driving mechanisms contain elements which define whether a certain game is present. The actor and interaction column specifies the main actions of and interactions between actors in each game. The strategic risks refer to game states that lead to bad outcomes for involved actors or the collective as a whole. Although these games have different characteristics, they can interact and exist at the same moment in time.

**Table:** Description of Games as Described in Earlier Policy and Game Theoretic Literature^22^

**Game**	**Driving Mechanisms **	**Actor and Interaction Dynamics**	**Strategic Risks**
Multi-Issue game	Interdependencies between issues and actors	It characterizes multiple actors having different incentives aim to reach consensus in a decision-making process that was in a deadlock in the first place	Deadlock situations block the decision-making; too many issues at the table create an overly complex situation that no-one can oversee; free fight
Cascade game	Time and hierarchical path dependency	It shows the tendency of intelligent actors, in case of uncertainties, to follow the decisions of others	Sub-optimal outcomes because of convergence in early phase; actors block the process at a certain level; decision based on incorrect information
Volunteers Dilemma	Free riding and wait-and-see behavior	It explains why one or more actors take the responsibility for the group to prevent a worst-case scenario from happening	No one volunteers leading to worst-case scenario; free riding; volunteer is not recognized; volunteer is blamed by others
Principal-Agent	Power and information asymmetry	It explains the power position of the subordinate, ie, the agent	Either actor acts strategically on the information or power asymmetry only for own benefit, collaboration breaks down
Hub-Spoke game	One actor leading the negotiations	It creates an incentive for inflated claims, the spokes can reach agreements among each other and create strategic issues for the hub	Spokes create strategic issues for the hub; delayed process because spokes are many and approached individually; limited learning
Diners Dilemma	Agreements made	It is beneficial to be the first one to violate the agreements made	No urgency to cooperate; once one actor violates the agreements other follow
Battle-of-the-Sexes	Conflicting interest and a common goal	It explains why one of the actors must adapt its decision to resolve the conflict	Conflict situation, no decision is made; suboptimal outcome due to compromise

###  Study Sample

 Interview participants were selected using snowball sampling, starting with actors currently involved in MBD policy-making. The set of actors currently engaged in developing and carrying out policy around MBD is relatively small. We therefore also aimed to include actors from MBD-relevant domains. For instance, considering the future of growing MBD risks, it may be relevant to include environmental actors that currently have no (active) role in MBD prevention or control. In total, 26 semi-structured interviews, with a duration between 60 and 90 minutes, were conducted between June 2021 and May 2022 with key stakeholders from several organizations (for details, see Supplementary file 1). The following subjects were discussed during the interviews (for interview guide, see Supplementary file 2): participant perspectives on MBDs policy in the Netherlands, key substantive and strategic uncertainties, and ways of handling these uncertainties. A second round of two validation interviews was performed in July 2022. Interviewees were selected based on them representing actors in at least two clusters of games and such that all clusters were covered at least once (See explanation about the clusters hereafter). During the validation interviews interviewees reflected on the games identified through the semi-structured interviews and how the identified games may evolve in the future.

###  Identifying Games and Game Clusters

 To identify games in the context of MBD decision-making, interview transcripts were according to the driving mechanisms of the seven game concepts (Table), adapted from earlier research.^27^ We identified a game as such when the driving mechanisms or action dynamics of a particular game were mentioned by at least two interviewees. We characterized the identified games by defining for each of them: the *actors *(who is playing the game), the *content *(what is the game about, what is at stake), and the *process* (how has the game developed over time and what actions were chosen proactively or in response to actual or anticipated actions by other actors). Subsequently, we clustered games with the same set of actors and overlapping content. Interactions between games in clusters were characterized by the dimensions of time and decision-making level (ie, operational, tactical, strategic). Games that did not fit into a cluster were excluded at this point. All transcripts were coded by two authors (HB and FB) independently. Ambiguities were resolved in discussions. Coding agreement was discussed across three sessions and author MB acted as referee in case of conflicts.Subsequently, strategic risks and policy recommendations were formulated based on comparing the study results (individual games and clusters of games) with game theoretic literature.

## Results

 In total, 15 games were identified. Some games were identified three times (Cascade game and Principal-Agent game), some were identified twice (Battle-of-the-Sexes game, Hub-Spoke game, Multi-Issue game, Volunteers Dilemma), and the Diners Dilemma was identified once. Across all games the Ministry of Health (MOH), municipalities and two large governmental institutions (National Institute for Public Health and the Environment [RIVM] and Netherlands Food and Consumer Product Safety Authority [NVWA]) were mentioned often as actors. The games were divided into three clusters, each with an increasing scope. The first cluster deals with the concrete issue of invasive mosquito control (Figure 1 and Table S2 in Supplementary file 3). This cluster is characterized primarily by a Volunteers Dilemma. MBD are zoonotic diseases, this is reflected in the second cluster that deals with zoonotic policy in the Netherlands (Figure 2 and Table S3 in Supplementary file 3). The central games in this cluster are a Cascade game and a Battle-of-the-Sexes game. The third cluster is the context in which the first two clusters take place. It concerns multi-level governmental decision-making in the Netherlands in general and is characterized by cascading Multi-Issue games that at times can evolve into Battle-of-the-Sexes games (Figure 3 and Table S4 in Supplementary file 3). Two games concerning water management (Principal-Agent and Cascade game) in the Netherlands and one about fragmentation in the broad municipal context (Multi-Issue game) did not fit into one of these three clusters and were excluded from the analysis.

**Figure 1:**
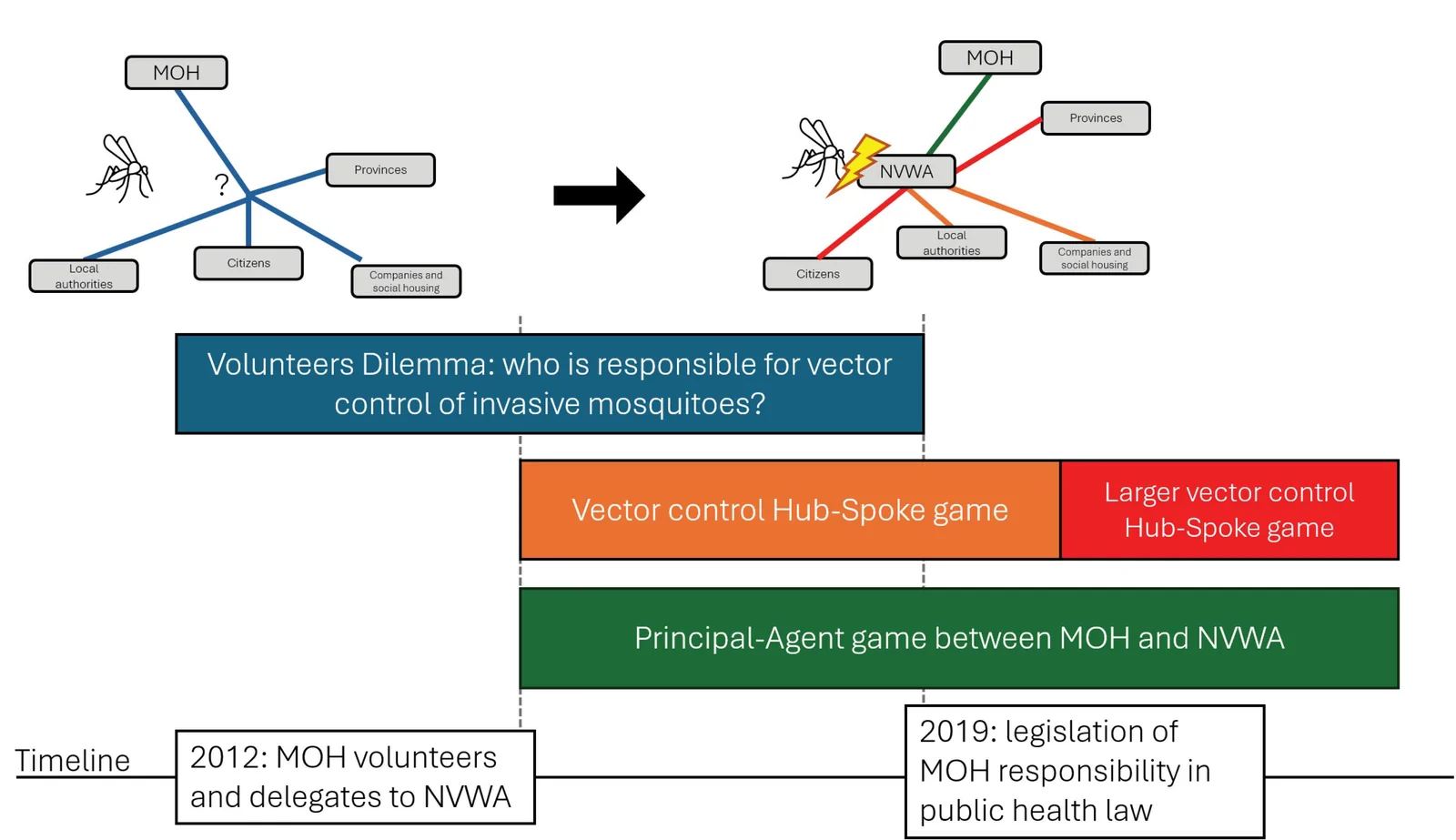
Conceptual Mapping of the Responsibilities Around Invasive Mosquito Control Cluster. The bottom part shows at which times which games were played, the top part shows the involved actor constellations. The lines between actors represent the played games. Vector control is depicted as a mosquito. Abbreviations: MOH, Ministry of Health; NVWA, Netherlands Food and Consumer Product Safety Authority.

**Figure 2:**
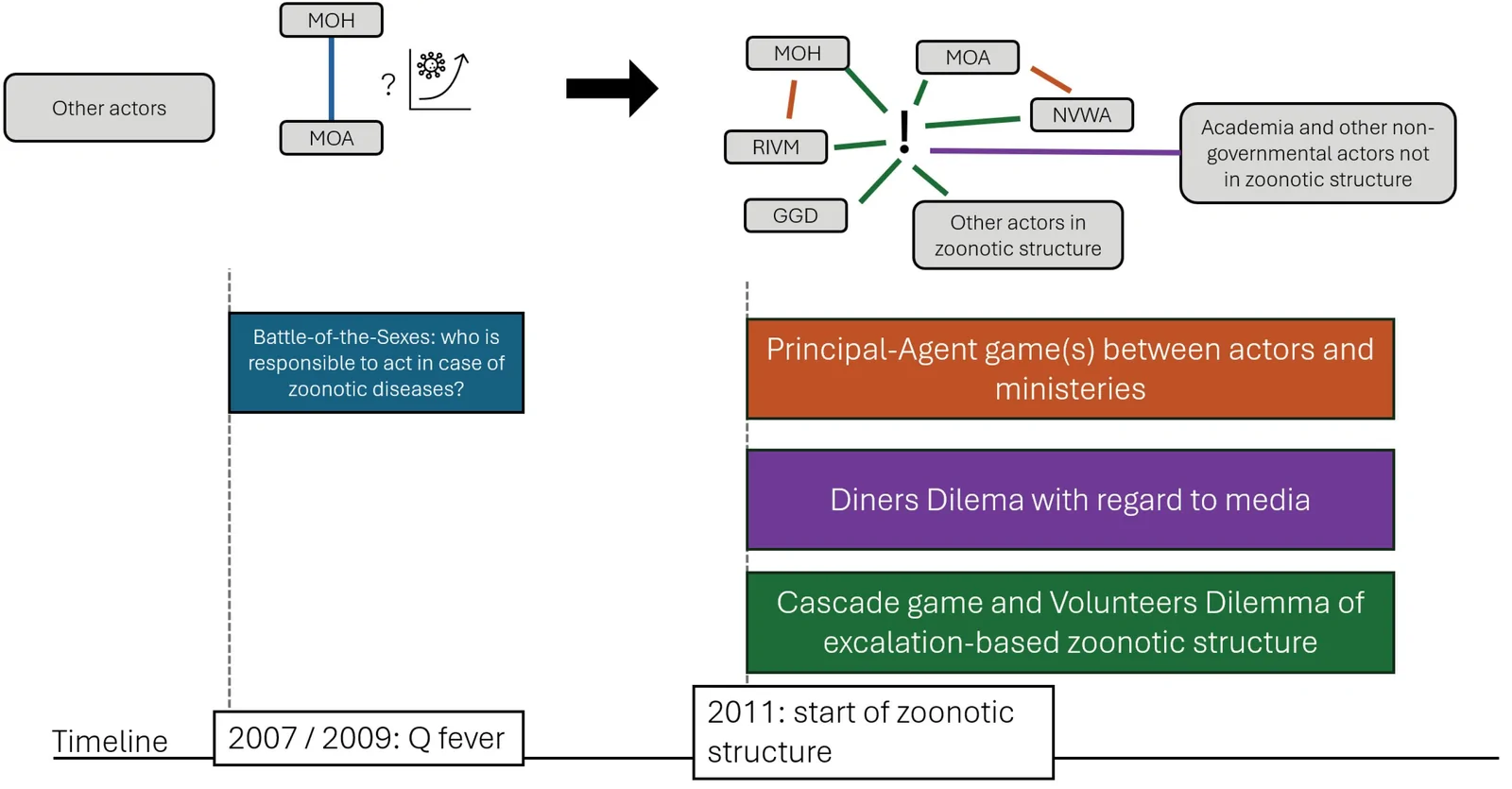
Conceptual Mapping of the Zoonotic Structure Game Cluster. The bottom part shows at which times which games were played, the top part shows the involved actor constellations. The lines between actors represent the played games. Abbreviations: MOH, Ministry of Health; NVWA, Netherlands Food and Consumer Product Safety Authority; RIVM, National Institute for Public Health and the Environment; MOA, Ministry of Agriculture; GGD, municipal public health service [Gemeentelijke Gezondheidsdienst].

**Figure 3:**
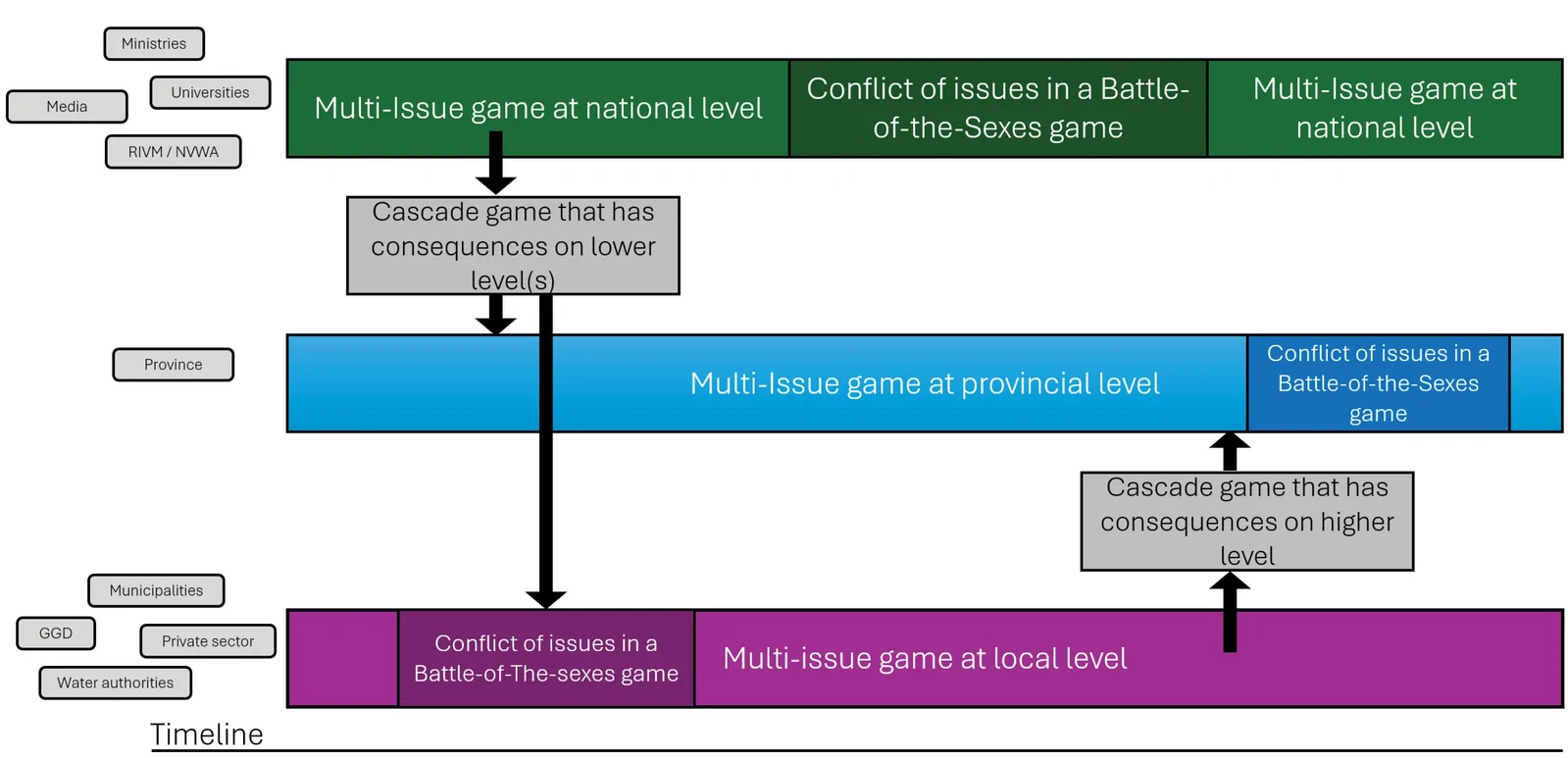
Conceptual Mapping of the Multi-level governance Cluster Across Hierarchical Levels and Time. The bottom part shows at which times which games were played, the top part shows the involved actors constellations. The lines between actors represent the played games. Abbreviations: NVWA, Netherlands Food and Consumer Product Safety Authority; RIVM, National Institute for Public Health and the Environment; GGD, municipal public health service [Gemeentelijke Gezondheidsdienst].

###  Game Cluster One: Responsibilities Around Invasive Mosquito Control

 Who takes up vector control to delay the introduction of invasive mosquitoes resembles a Volunteers Dilemma. The actors in this game are the MOH, provinces, municipalities, government agencies such as NVWA and RIVM, and companies/citizens that can accidentally import invasive mosquitos. In general, since vector prevention or control is costly, actors in this dilemma are pushed towards free riding. An example of this game is a situation when the eggs of *Aedes albopictus* (Asian tiger mosquito) are brought into the Netherlands with imported tires. Municipalities and tire companies do not have a direct financial incentive to take preventative action (eg, stop tire imports or invest in dry storage):

 “*Our policy objective is to prevent the establishment of exotic mosquitoes for as long as possible. [A MOH mandate] was then the only solution to guarantee that. Otherwise it would remain the responsibility of the municipalities, and those municipalities — and this also emerged from an evaluation — would still rather think about [safeguarding] employment. [They] do not want to impose extra requirements on tire companies such as creating additional roofing. Because those coverings are of course not free, and [municipalities are] not going to enforce that. So we then chose to arrange it by law” *[MOH interviewee].

 The possible political rewards of having prevented an outbreak are uncertain and probably beyond the current electoral cycle, while the costs are visible and immediate. All of this is characteristic of a classic “prevention paradox” as the “reward” occurs elsewhere or on a long-term horizon. The MOH has responded to this by centralizing invasive mosquito control responsibility, becoming the “volunteer.” Since 2012 MOH (through the NVWA) has conducted vector control, a role which was later legislated in 2016.^28^ Any mosquito sightings are reported by the spokes (companies or citizens) to the hub (NVWA) who is responsible for vector control. This centralization gives rise to Hub-Spoke game dynamics:

 The two games in this cluster are related because the volunteer in one is the hub in the second. The “volunteer hub” NVWA cannot act independently because it needs information from the spokes about mosquito findings. These may be reluctant to provide information because of fear of stigmatization or the arduous vector control process, in which NVWA pays them multiple visits and they have to continually empty water containers in their back yard:

 “*Contact with citizens is actually positive. That may also have to do with the fact that this is a relatively new problem here, so it is still new when the inspector comes by and checks the gardens, and the participation and the results of what the citizens think of it are positive and good. […] But we also know that if problems persist and we come for the third year in a row because a company has again imported a tiger mosquito, communication can then also be more difficult in keeping citizens positively involved” *[NVWA interviewee].

###  Game Cluster Two: The Zoonotic Structure

 Like for other zoonotic diseases, the prevention and response policy to MBDs affect multiple domains, primarily those of human health, animal health, and environmental health; but extending into the broader domains of spatial/urban planning, water management, and agricultural policies. A good example of possible dynamics is the 2007 Q-fever outbreak in the Netherlands.^29,30^ The problem originated in and could be acted upon in the animal health domain, but the disease burden was in the human health domain. Because there were substantive uncertainties (eg, transmission routes between birth-giving goats and humans), it was not immediately clear which ministry should take the lead: Health or Agriculture. Moreover, since ministries are at the same hierarchical level, negotiations led to delays and discussions:

 “*During Q fever the zoonosis structure did not yet exist, and then you get ministries positioned opposite each other, and then you are at the same level, so who takes the lead? […] The interests of one are always greater than those of the others […]. It was very interesting that the disease burden was on the people and the solution with the [Ministry of Agriculture], then you really have misery, because the one who has to take action and for whom it costs the most is not the one who has to provide the solution”* [RIVM interviewee].

 This dynamic can be characterized as a Battle-of-the-Sexes game. Both ministries shared the goal of maximizing welfare in the Netherlands, but defined this differently (eg, well-being versus economic margin). The consequences of this game, with 116 deaths between 2007-2022 and more than four thousand chronic Q-fever patients, pushed the two responsible ministries to develop the “zoonotic structure” in 2011 to reduce the chance of ending up in a battle-of-the-sexes dynamic. The zoonotic structure regularly brings together experts from the animals and human domains. It is based on signals of potential zoonotic threats and there are explicit escalation protocols. The structure is more expert-led for low severity signals (eg,invasivemosquito settlement in southern Europe) and more politically led for high severity signals (eg, outbreak among humans in the Netherlands).

 The main game observed in the zoonotic structure is a Cascade game where the aim is to prevent escalation of signals to higher severity levels. Regularly bringing together experts from the two domains helps to build mutual trust that can prevent the disruptive impact of the Battle-of-the-Sexes dynamics. Another identified game is the Principal-Agent game between the power-wielding actors (ministries) and the information-wielding actors (RIVM and NVWA). The power and information asymmetries between the two actors have been dealt with by building mutual trust between the people responsible.

 “*[The zoonosis structure] has been in place for well over ten years. As a result, we have regular contact moments, but also a lot of informal contact. The signaling meeting takes place every month, and the following week we always have a meeting with [The ministry of Agriculture] and the RIVM to discuss new issues, new signals, and various ongoing matters. We also draft a lot of documents together, or respond to parliamentary questions. In the past we even had a weekly ‘zoonosis lunch.’ Sure, sometimes there is friction over the content, but we’re colleagues. The lines of communication are short. We don’t always want the same things, but that’s logical because we all have different interests, and it’s up to us to work through that properly” *[MOH interviewee].

 Protocolization, however, also means that only particular organizations have a reserved seat at the table. Since some signals originate from outside of these organizations, there are (informal) process agreements with those providing notifications, eg, academia or the private sector. A Diners Dilemma is present when the incentives for those “outside actors” deviate from those of governmental actors, for example when academia seeks media attention for research findings. For universities this is an important part of creating research impact and attracting future funding, but some media messages may be seen as too alarmistic by governmental actors:

 “*Another important group are the mosquito researchers, those running research programs. They naturally also have ‘interests’ in putting the issue more on the agenda in order to attract funding. They are valued colleagues and they do solid research, but they also sometimes make statements about human health risks where we think: this is not really your role” *[MOA interviewee].

 Finally, the zoonotic structure gives room for a Volunteers Dilemma like the one described in cluster 1 for invasive mosquitoes. Some authorities with a mandate across several domains (eg, municipalities or provinces) might be pushed to free-ride more than those with a specific MBD-related mandate (eg, RIVM or NVWA). The dangerous events here are disease outbreaks that could have been prevented, and ministries step in to organize preparedness and response. The final game in this cluster concerns policy-making around native mosquito’s where municipalities carry the legal responsibilities but lack the knowledge to deploy vector control. It is unclear how this game would interact with the other games in this cluster exactly. The response to West Nile virus, which is transmitted by native mosquitoes, was taken up by actors in the zoonotic structure (eg, RIVM, Center for Monitoring of Vectors at NVWA, etc) as well as the response to COVID-19. Lessons from these experiences are still being learnt and will most likely impact the way MBDs are handled in future. In the validation interviews it was expected that the underlying Battle-of-the-Sexes game between human health and animal health will be broadened and include issues from the area of the living environment.

###  Game Cluster Three: Multi-level Governance

 Three important connected issues in Dutch politics relate to MBDs: climate adaptation, infectious disease control/prevention and biodiversity. These issues are acted upon across large time scales and across various government levels: national, provincial and municipal. This creates an overarching Multi-Issue game for each level in the third cluster. When incentives conflict, a Battle-of-the-Sexes game can occur between actors at a particular governmental level. To navigate or circumvent the Multi-Issue game, actors may introduce new issues to broaden the solution space. Examples of issues brought to the table to increase the sense of urgency are: COVID-19 circumstances, ticks, oak processionary moth, nature-based solutions, land use and urbanization, the housing crisis, and the use of pesticides.

 “*We all want more biodiversity, but we certainly don’t want more mosquitoes. In the city you quickly end up getting in each other’s way with these kinds of issues”* [Municipality interviewee].

 Complicating these Multi-Issue games is the fact that there is hierarchical dependency between them: eg, the outcome of a national level game can define the possible future actions provinces and municipalities can take. There can be tension between local-level actors that may desire a narrow solution space (as they have to balance multiple objectives) and national-level actors that may prefer a wider, more flexible, solution space:

 “*[Our need] is raising awareness [that] we are already dealing with [mosquitoes] now, and that it will get worse in the future because of climate change. What are we talking about? What are the health risks? And then comes the important follow-up: ‘and then you can do this and that, and it costs this much.’ If [we] take it into account in the design phase, [we] can build a stable and sustainable system without much extra expense” *[Local water authority interviewee].

 The process of negotiating solution space across levels takes time and requires a balance between cost-effectiveness and broad support. Preventative action is most cost-effective in an early stage long before MBD outbreaks but gaining strategic momentum for action is easier during or after an MBD outbreak.

## Discussion

 This study identified 15 games (grouped across 3 clusters) around MBD decision-making in the Netherlands. Control of invasive mosquitos that may in the future carry MBDs is taken up by national-level stakeholders (cluster 1). Surveillance for and response to MBD outbreaks shows the dynamics of the zoonotic game structure (cluster 2). These two clusters are part of multiple Multi-Issue games that take place across time and governmental levels (cluster 3).

###  Strategic Uncertainties

 Reflecting on these results, we observed that many interviewees expressed the feeling of being uncertain about who ‘owns’ the problem of MBD policy, especially prevention. Many actors who played games in clusters 1 and 2 did not see MBDs as an urgent problem yet, especially those not currently involved in the zoonotic structure or invasive mosquito control. This made them experience strategic uncertainty: they did not know if they can act, are obligated to act and what other actors were going to do: MBD decision mandates are therefore not always clear (Volunteers dilemma). And if actors did see actions they could take, they were often unsure about a timing that would benefit their priorities best in a cost-effective manner (Cascade games, Battle-of-the-Sexes games). The core strategic uncertainties are therefore lack of problem ownership and the prevention paradox. Although our results show that the Volunteer’s Dilemma and Battle-of-the-Sexes games are central to understanding strategic uncertainty in MBD policy, many other games are played across different actor constellations and time horizons. Importantly, many of these games have only a little to do with MBDs as such: MBD policy overlaps with zoonotic policy in general and wider societal policies on topics such as climate change adaptation: the Multi-Issue games of cluster 3.

 The strategic uncertainties were observed to be conditional on substantive and institutional uncertainties. On the one hand, substantive uncertainty makes it harder for actors to determine their own role. In fact, part of the strategic uncertainty comes from unanswered questions about MBDs that preclude national-level stakeholders from developing the concrete boundaries local-level stakeholders need. On the other hand, institutional uncertainty bounds actors’ perspectives and modes of internal and external communication. It is an open question to what extent actors are aware of (the consequences of) games they are playing. This could also be characterized as institutional uncertainty: more science/information-focused actors may focus mostly on substantive uncertainty with the idea that if all substantive uncertainty is gone, the other types of uncertainties will resolve themselves. Political actors are more likely to be aware of strategic and institutional uncertainty but are less able to judge the extent of substantive uncertainty.

###  General Recommendations

 Navigating strategic uncertainties in a way that minimizes strategic risks requires involved actors are made aware of games they participate in as well as related game (clusters) they do not directly participate in. With such awareness they can better anticipate what might happen and communicate with other actors to reduce strategic risks. However, learning opportunities in everyday practice focus mostly on substantial uncertainties rather than strategic uncertainties. Moreover, these practices often remain within institutional networks rather than transdisciplinary networks. Therefore, a new perspective on policy learning and adaptation centers around working *with* substantive uncertainties instead of focusing only on uncertainty reduction. This involves practices of collaborative, reflexive monitoring in deliberative dialogues among actors involved in an ongoing cycle of policy adaptation and revision.^31,32^ A more inclusive approach would leverage the sources and experiences of people in places where mosquitos occur, increase place-based awareness and a sense of urgency, and the possibility of resolving citizen or business concerns and barriers to mosquito notifications and joint action.

###  Recommendations Per Cluster

 In the first cluster (*responsibilities around invasive mosquito control*), the central game is the Volunteers Dilemma, ie, who should act to delay and manage the introduction of invasive mosquitoes. This game seems to be resolved for all actors involved since NVWA conducts the vector control. However, there is the risk that the worst-case scenario (permanent mosquito settlement) happens because there is little knowledge or incentive for other actors to take preventative action. In time, the problem may grow beyond a size that the NVWA can handle on its own. Incentives (eg, education, subsidies or fines or regulations) can help spoke-actors to take preventative action to alleviate the hub-actor. Fines, though, may deter citizens from reporting mosquito encounters. To deal with the increasing pressure on the NVWA in case of more invasive mosquito imports, we recommend a multi-level system, for example with regional focal points for invasive mosquitoes.

 In the second cluster (*the zoonotic dilemma and structure*), the formulation of the zoonotic structure can be seen as a response to the risk of future Battle-of-the-Sexes dynamics. There is a risk that the protocolization between the human health and animal health domains, in an effort to reduce Principal-Agent related strategic risks, overlooks (small) actors from other domains such as the environmental domain.^12^ The trust that is built depends on personal relationships that take time to form, making the relationships between actors fragile when people retire or change jobs. It is therefore recommendable to have multiple ties between organizations, on both the protocol and person-to-person. Another risk, related to the Diners Dilemma, is that actors outside the structure, for whom only (possibly informal) process agreements exist (eg, around information sharing or communication), violate these agreements or free ride to pursue their own objectives. A way to deal with this risk is to discourage free riding by broadening the dangerous event from the impactful but rare “outbreak among humans” to more politically valuable things like “biodiversity,” “mosquito nuisance,” or “sleep disruption.”

 The third cluster (*the governmental process across levels*) is characterized by interactions between the Multi-Issue game, the Cascade game and the Battle-of-the-Sexes games. A strategic risk in this cluster is that the Multi-Issue game turns into an overly complex situation where too many issues and interdependencies lead to no single actor overseeing the whole. Instead of adding more and more issues to the table, Joint workshops may help identify and define strategies to navigate the various issues of the Multi-Issue game. Moreover, it may show which new actors may be necessary to involve to address or resolve conflicts, such as actors from the environmental domain with whom little collaborations exists on this topic.^12^ A Battle-of-the-Sexes game situation can be addressed through coupling of multiple issues to broaden the solution space and make it part of the broader Multi-Issue game. When coupling of issues is not feasible, appointing a jointly agreed-on independent referee can help to resolve the conflict. Appointing this referee beforehand and informing actors about this referee is a way to anticipate the situation. A final strategic risk is that the outcomes of the three games in this cluster align most with actors’ short-term objectives because these are more visible for the actors involved. Short-term outcomes are more likely to provide strategic political benefits but also to give rise (even more) to Battle-of-the-Sexes games. Furthermore, prevention is a long-term process where gains are not immediately (or ever) visible. In designing or evaluating MBD policy, we therefore recommend including process criteria, such as stakeholder commitment and extent of collaboration. Finally, we recommend, where possible, decoupling MBD considerations from the short-term focused political process.

###  Transferability of Recommendations

 The extent to which the cluster-specific policy recommendations are useful for actors outside of the Netherlands depends on the local MBD context and actor constellations and is different for each game cluster. Other countries where invasive mosquitoes have not yet settled are likely to encounter a Volunteers Dilemma much like our first game cluster. Although Battle-of-the-Sexes dynamics between the human health and animal health domain are likely encountered in more countries, the dynamics in the second game cluster are mostly due to specific policy solution (the zoonotic structure) pursued in the Netherlands. Finally, although the third cluster is specific to the Netherlands’ governmental hierarchy, issues that are dealt with (climate change, biodiversity, etc) are probably relevant in many other countries. In fact, it could be argued that there could be a fourth “European Union level” positioned above all nation-specific governmental hierarchies.

###  Strengths, Limitations, and Future Research

 This study contributed to the discourse around MBD policy in the Netherlands in various ways. It is a novel application of game theory that provides insight into the strategic uncertainties experienced by actors in and around MBD policy, and how actors deal with these uncertainties in the context of sometimes contradictory institutional incentives. There are strategic uncertainties that make substantive evidence-based decisions feasible only if the evidence fits within the implicit contextual framework of underlying motives and institutional constraints. In this study, the focus on the interaction between games is novel, expanding the game structures used to characterize dynamics in multi-actor decision-making. We find that such strategic uncertainties could be mapped well to archetypal games and shows that the selected game concepts from earlier studies in public decision-making for infrastructure policy and other policy domains also exist in public health decision-making.By bridging the fields of public health and game theoretic research in the area of multi-actor decision-making, the results become also practically useful for policy-makers. Following up on the recommendations to address strategic uncertainties is a first step towards using game theoretical concepts to support real-life decision-making.^21^

 The primary limitation of this study is that the interviews were not designed specifically to elicit information on games but instead focused on substantive and strategic uncertainty. This may imply we were unable to fully capture all relevant (elements of the) games. Another limitation is the fact that we considered seven games. The reason for selecting these games is based upon an earlier study^27^ where a taxonomy of games is used to select a set of games that cover a set of diverse decision-making situations. These situations include both 2-player and multiple-players, cooperative and competitive behavior, and hierarchical and network relations to name a few distinctions. The selection of games has been used to characterize strategic uncertainty and support decision-making in other domains like long-term infrastructure planning.^27^ As a consequence, the selection of game archetypes may have resulted in certain policy dynamics falling outside the identification. The inherent domain-spanning nature of MBDs also makes it so that the games identified in this study are not just about MBDs, but also about related (and very dynamic) topics such as zoonotic disease in general and climate change adaptation.

 A consequence of choosing a game theoretic approach in studying strategic uncertainty is that we have made assumptions about the rationality of actors in decision-making. While in reality actors rationality is bounded by psychological and institutional factors, a classical game theory perspectives assumes that actors are fully rational.^33^ In practice, people make use of biases and heuristics to make decisions^34^ and institutional path dependency plays an important role in strategic decisions.^35^ These elements play an important role in explaining how decisions are made beyond the rationality assumptions of game theory. This study has not focused on these explicitly, however, by including games from both game theory and public administration sciences we tried to overcome this. For example, institutional path dependency is reflected in the Cascade game.^36^ Biases in decision-making could lead to irrational or suboptimal outcomes in game theoretical terminology. In this study, we did not aim to calculate optimal outcomes because it would require making assumptions about the behavior of actors we could not observe or question. Instead, we qualitatively describe different possible outcomes of the games and use the games to explain strategic uncertainty in the context of MBD. For future research it would be interesting to include these concepts in the analysis to better understand how biases influence decisions. Moreover, future research could include actors directly, at multiple points in time, in the identification of games and evaluation of recommendations (see eg, Bekius and Gomes^21^).

## Conclusion

 MBD is afflicted with a prevention paradox where prevention measures have immediate costs but intangible future benefits. Actors in or adjacent to the MBD policy domain experience strategic uncertainty because there is a lack of clear problem ownership. Policy thus requires intersectoral and interdisciplinary coordination. This strategic uncertainty was observed in three game clusters around invasive mosquito control, the zoonotic coordination structure, and multi-level governance of MBDs. These reveal ambiguity in the boundaries of strategic, substantial and institutional uncertainties surrounding MBD policies as well as fundamental interdependencies between actors, their objectives, and their (lack of) actions.

 Strategic uncertainty around MBD policy requires a governance approach that incentivizes intersectoral and integrative policies, bringing together the expertise and organizational capacities of all involved actors and domains. Governments and policy sectors are nevertheless in many countries organized in pillared subsystems with delegated power to executive agencies, which operate at a distance of political decision-making. This, coupled with institutional incentives such as departmental and sectoral accountability norms and requirements pose institutional and subsequently strategic uncertainty for cross-sectoral coordination and collaboration. Administrative reforms attempting to increase policy coordination and integration across different countries are associated with left wing political parties.^37,38^ The current shifts to (radical) conservative right governments across different continents thus might imply that policy integration to resolve or mitigate climate-related (potential) health risks such as MBDs is not likely to occur. In the likely absence of political decisions to facilitate policy coordination and integration in the near future, our study contributes to enhanced strategic awareness and capacity in MBD prevention and control. Insight into strategic uncertainties could enable actors to collaboratively design new governance strategies that can help to articulate, anticipate, and mitigate strategic risks. We propose a policy and governance approach for MBD prevention that combines the mitigation of both substantive and strategic uncertainties. Such mitigation might likely be achieved through collaborative, reflexive monitoring in deliberative dialogues among stakeholders involved in an ongoing cycle of policy adaptation and revision.^31,32^ Deliberate change in strategic behavior may change the course of the games towards more quick, more integrative and more impactful prevention of MBDs.

## Acknowledgements

 We thank the interviewees for taking the time to talk to us.

## Disclosure of artificial intelligence (AI) use

 Not applicable.

## Ethical issues

 The research was approved by the social sciences ethical committee of Wageningen University and Research.

## Conflicts of interest

 Authors declare that they have no conflicts of interest.

## Data availability statement

 The data underlying this article are interview transcripts. These cannot be shared publicly due the privacy of individuals that participated in the study.

## Supplementary files


Supplementary file 1. Overview of Interviewed Stakeholders.

Supplementary file 2. Interview Guide.

Supplementary file 3 contains Tables S2-S4.
