# Supplementary material for: Strategic Uncertainties Surrounding Mosquito-Borne Disease Policy-Making in the Netherlands: A Game Theoretic Analysis
Source: Int J Health Policy Manag. 2026 Jun 29;15:9091. doi: 10.34172/ijhpm.9091 (PMC13729732; doi:10.34172/ijhpm.9091)
Supplement: Supplementary file 1 [file ijhpm-15-9091-s001.pdf]

Article title: Strategic Uncertainties Surrounding Mosquito Borne Disease Policy-Making in the Netherlands: A Game Theoretic Analysis

Journal name: International Journal of Health Policy and Management (IJHPM)

Authors' information: Henk Broekhuizen[1,2]*, Femke Bekius[3], Pauline A. de Best[4,5], Marleen Bekker[1]

[1]Health and Society Group, Department of Social Sciences, Wageningen University and Research, Wageningen, The Netherlands.

[2]Department of Epidemiology, Health Promotion, and Policy Advice, GGD Twente, Enschede, The Netherlands.

[3]Department of Methodology, Nijmegen School of Management, Radboud University, Nijmegen, The Netherlands.

[4]Department of Viroscience, Erasmus Medical Center, Rotterdam, The Netherlands.

[5]National Institute for Public Health and the Environment (RIVM), Bilthoven, The Netherlands.

***Correspondence to:** Henk Broekhuizen; Email: h.broekhuizen@ggdtwente.nl

Citation: Broekhuizen H, Bekius F, de Best PA, Bekker M. Strategic uncertainties surrounding mosquito borne disease policy-making in the Netherlands: a game theoretic analysis. Int J Health Policy Manag. 2026;15:9091. doi:10.34172/ijhpm.9091

Supplementary file 1. Overview of Interviewed Stakeholders

Table S1. *list of institutional affiliation of interviewees and their main field of expertise. The third columns reports the domains the interviewees are mostly active in (H=human health, E=environment, A=animal health, V=vectors) and the fourth columns reports the governmental level where they mostly function (N=national, P=provincial, L=local). The institutional abbreviations stand for the following. RIVM = national institute for public health and the environment, GGD = municipal public health services, PBL = Netherlands environmental assessment agency, LNV = ministry of agriculture, nature and food quality, WUR = Wageningen university and research, NVWA = Netherlands food and consumer product safety authority, CMV = centre for monitoring of vectors, VWS = ministry of health, welfare and sport, DWHC = Dutch wildlife health centre.*

Institution	Field	Domains	Level
RIVM	1 medical entomologist, 1 hydrologist, 1 biologist, 1 medical doctor	H, E	N
GGD	1 nurse, 2 medical doctors, 1 entomologist	H, E	L
Safety region	1 manager, 1 risk analysist	H	L
PBL	1 researcher	E	N
Water authority	3 policy advisors	E	L
LNV	1 policy advisor	A	N
Pest control company	1 technical manager	V	N
Municipality	1 water expert, 1 ecologist, 2 policy advisors	E	L
WUR	1 entomologist, 1 ecologist	V, E	N
NVWA	1 veterinarian, 2 entomologist (from NVWA-CMV)	A, V	N
VWS	2 policy advisors	H	N
Province	1 entomologist, 1 policy advisor	E	P
DWHC	1 pathologist	A	N
