# Supplementary material for: Strategic Uncertainties Surrounding Mosquito-Borne Disease Policy-Making in the Netherlands: A Game Theoretic Analysis
Source: Int J Health Policy Manag. 2026 Jun 29;15:9091. doi: 10.34172/ijhpm.9091 (PMC13729732; doi:10.34172/ijhpm.9091)
Supplement: Supplementary file 2 [file ijhpm-15-9091-s002.pdf]

Article title: Strategic Uncertainties Surrounding Mosquito Borne Disease Policy-Making in the Netherlands: A Game Theoretic Analysis

Journal name: International Journal of Health Policy and Management (IJHPM)

Authors' information: Henk Broekhuizen[1,2]*, Femke Bekius[3], Pauline A. de Best[4,5], Marleen Bekker[1]

[1]Health and Society Group, Department of Social Sciences, Wageningen University and Research, Wageningen, The Netherlands.

[2]Department of Epidemiology, Health Promotion, and Policy Advice, GGD Twente, Enschede, The Netherlands.

[3]Department of Methodology, Nijmegen School of Management, Radboud University, Nijmegen, The Netherlands.

[4]Department of Viroscience, Erasmus Medical Center, Rotterdam, The Netherlands.

[5]National Institute for Public Health and the Environment (RIVM), Bilthoven, The Netherlands.

***Correspondence to:** Henk Broekhuizen; Email: h.broekhuizen@ggdtwente.nl

Citation: Broekhuizen H, Bekius F, de Best PA, Bekker M. Strategic uncertainties surrounding mosquito borne disease policy-making in the Netherlands: a game theoretic analysis. Int J Health Policy Manag. 2026;15:9091. doi:10.34172/ijhpm.9091

Supplementary file 2. Interview Guide

Goals: We would like to better understand the central issues of organization around mosquito-borne diseases and coordination between all parties involved in the Netherlands.

Block 1: Building relationship and trust, maximizing storytelling

Question 1 (intro general): Can you tell us something about your daily work? What does your day look like?

Question 2 (intro mosquito diseases): How is your work related to mosquito-borne diseases?

Block 2: Going into depth

Question 4 (substantive uncertainty): How do you deal with open questions or information needs you have regarding policy making or implementation on mosquito-borne diseases?

Question 5 (priorities): What are the most important goals/priorities you have regarding mosquito-borne diseases?

Question 6 (decisions): Can you give us examples of how decisions are made on a complex issue for which there is not an immediately obvious solution?

Question 7 (dependencies): Can you tell us something about how you collaborate with others on mosquito-borne diseases?

Question 3 (problem perception): What do you think are the biggest organizational/coordination challenges facing the Netherlands regarding mosquito-borne diseases? What makes it complicated?

Question 8 (tools): How are you trying to make the issues manageable? What is in the "toolbox" of coordination tools?

Block 3: crucial/sensitive issues

Question 9 (frustrations): Have there been times when things really just didn't work out? Can you give examples of this?
