# Supplementary material for: Strategic Uncertainties Surrounding Mosquito-Borne Disease Policy-Making in the Netherlands: A Game Theoretic Analysis
Source: Int J Health Policy Manag. 2026 Jun 29;15:9091. doi: 10.34172/ijhpm.9091 (PMC13729732; doi:10.34172/ijhpm.9091)
Supplement: Supplementary file 3 [file ijhpm-15-9091-s003.pdf]

Article title: Strategic Uncertainties Surrounding Mosquito Borne Disease Policy-Making in the Netherlands: A Game Theoretic Analysis

Journal name: International Journal of Health Policy and Management (IJHPM)

Authors' information: Henk Broekhuizen[1,2]*, Femke Bekius[3], Pauline A. de Best[4,5], Marleen Bekker[1]

[1]Health and Society Group, Department of Social Sciences, Wageningen University and Research, Wageningen, The Netherlands.

[2]Department of Epidemiology, Health Promotion, and Policy Advice, GGD Twente, Enschede, The Netherlands.

[3]Department of Methodology, Nijmegen School of Management, Radboud University, Nijmegen, The Netherlands.

[4]Department of Viroscience, Erasmus Medical Center, Rotterdam, The Netherlands.

[5]National Institute for Public Health and the Environment (RIVM), Bilthoven, The Netherlands.

***Correspondence to:** Henk Broekhuizen; Email: h.broekhuizen@ggdtwente.nl

Citation: Broekhuizen H, Bekius F, de Best PA, Bekker M. Strategic uncertainties surrounding mosquito borne disease policy-making in the Netherlands: a game theoretic analysis. Int J Health Policy Manag. 2026;15:9091. doi:10.34172/ijhpm.9091

Supplementary file 3

Table S2: individual games as identified through the interview analysis in the 'responsibilities around invasive mosquito control' cluster. VD=volunteer's dilemma, HS=hub-and-spoke game. MOH=ministry of health, NVWA= Netherlands food and consumer product safety authority, GGD=municipal public health service.

Game	*Actors*	*Content*	*Process*
VD	MOH, NVWA, provinces, municipalities, companies, citizens	Dangerous event = settlement of invasive mosquito	MOH volunteers, formalized in public health law.
HS	Municipality = hub, spokes = depts. At municipality, NVWA,	Presence of invasive mosquito's, but actually wider:	Efficient working esp. at scale but more collaboration required between spokes in different domains.

	GGD, water authorities, social housing associations	questions about healthy living environment	
HS	NVWA = hub, citizens and companies = spokes	Presence of invasive mosquito's	Shift of entry method of invasive mosquitos from, issues surrounding privacy and stigma.

Table S3: individual games as identified through the interview analysis in the 'the zoonotic dilemma and structure' cluster. BS=battle-of-the-sexes game, CG=cascade game, VD=volunteer's dilemma, DD=dinner's dilemma, MOH=ministry of health, MOA=ministry of agriculture, RIVM=National institute for public health and the environment, NVWA= Netherlands food and consumer product safety authority, WUR=Wageningen university and research, OHP=One health pact

Game	*Actors*	*Content*	*Process*
BS	MOH and MOA	MBDs cross domains of both animals and humans	Protocolized zoonotic structure as response to difficulties during Q fever outbreak. Decisions made after joint deliberation. Responsibilities and roles protocolized.
CG	Actors in the zoonotic structure	Zoonotic structure processes	Preference not to escalate, protocol-driven culture, focus on animal and human health domains.
PA	MOH=principal, RIVM=agent, NVWA=agent	Roles in zoonotic structure and invasive mosquito control	Mutual professional trust, clear delineation of tasks, forming of professional 'elite'.

VD	RIVM, MOH, citizens, municipalities	Dangerous event: outbreak could have been prevented	Coupling to zoonotic structure cascade game, "waiting" for human cases, communication by both policy actors and academic actors (zoonotic structure dinners dilemma).
DD	RIVM, NVWA, MOH, WUR/OHP	Process agreement: align communication and information	Academia generates media attention that can have negative consequences for governmental actors.
PA	MOH+MOA =principal, municipalities=agents	Native mosquito policy	Municipalities carry responsibility, MOH has a role to put the topic on the agenda.

Table S4: individual games as identified through the interview analysis in the 'the governmental process across levels' cluster. MI=multi-issue game, BS=battle-of-the-sexes game, CG=cascade game, MOH=ministry of health, MOA=ministry of agriculture, RIVM= National institute for public health and the environment, GGD=municipal public health service

Game	*Actors*	*Content*	*Process*
MI	Public sector	Core issues: climate change adaptation, infectious disease prevention, and biodiversity.	Consensus building, coalition forming, tension narrowing/widening solution space.
BS	National: MOH, MOA, RIVM;	Friction between climate change adaptation and risk	Balancing act, cooperation is needed. Plays at different levels.

	Provincial: province; local: Municipality, GGD, water authority	of MBD. Overarching goal: healthy living environment	
CG	Public sector	Public policy on prevention	Prevention paradox, tension between clear rules and flexible scenario thinking. Long time between conception of policy and its execution.
